# Supplementary material for: Epidemic efficacy of Covid-19 vaccination against Omicron: An innovative approach using enhanced residual recurrent neural network
Source: PLoS One. 2023 Mar 24;18(3):e0280026. doi: 10.1371/journal.pone.0280026 (PMC10038250; doi:10.1371/journal.pone.0280026)
Supplement: Attachment — Submitted filename: Response Sheet omicron.docx [file pone.0280026.s001.docx]

**Response Sheet**

Dear reviewers,

We are thankful to you for reading our manuscript and providing valuable suggestions to improve it. The response with respect to queries asked by reviewers is mentioned below and highlighed in the manuscript.

**Reviewer #1**

**Question 1:** The structure of the of introduction can be improved. It is suggested to remove subsections of section 1 for an integrated text. The aim of the paper has been stated before section 1.1, and again, in section 1.2 and 1.3.

**Response:** The structure of Introduction has been changed and the sub-sectioning has been removed for an integrated text.

**Question 2:** Please check the journal guidelines whether the first letter of the authors’ names should be written in the citations within text, or not. A modification maybe required.

**Response:** The author’s names have been updated according to requirements

**Question 3:** The Comparison and reliability related results cab be extended. More accurate and competing methods should be further utilized in the result comparison. The prediction of basic ResRNN is more accurate than AR, GR, VAR. So, using these methods for comparison is not wise.

**Response:** The data has been trained on two more models namely, ARIMA and FB Prophet which are quite competing methods in time-series prediction and its result graphs have been added (refer Fig 4)

**Question 4:** Identify the names of the axis in Fig.4.

**Response:** The names of the axis in Fig 4(Now Fig 5) have been added

**Question 5:** The models can also be compared using the Mean Squared Errors.

**Response:** The Mean Squared Error (MSE) for the proposed model and some other models has been added.

**Question 6:** Its better to present the prediction results (such as Fig. 6) considering the competitive results of the other methods.

**Response:** The Fig 6 (Now Fig 7) is an Exploratory Data Analysis and comparision of this with other model is not applicable.

**Question 7:** It is suggested to add the pseudo code of the proposed method.

**Response:** We can provide the code as per requirement after acceptance.

**Reviewer #2**

**Question 1:** The language needs proofreading and please provide a proof if you already done this.

**Response:** The entire manuscript has been proofread carefully and the changes have been highlighted.

**Question 2:** In 1.1 how would you proof that other previous works are limited? Could you name any research?

**Response:** Most of the work regarding to COVID-19 has been done is classifying whether a person has COVID-19 or not using images. There are few articles on predicting COVID-19. Moreover, the architecture designed by us using residual architecture with LSTMS and GRU units has not been implemented yet.

**Question 3:** In 1.3 what do you mean by contribution? change the title or give more clear details please.

**Response:** The sub-sections in introduction has been removed as guided by reviewer 1.

**Question 4:** Have you reviewed any previous literature regarding omicron against Covid-19 vaccination? I could not find any. Please name some or highlight them for me.

**Response:** Yes, we have reviewed article for omicron against covid-19 vaccination and it has been highlighted in references section

**Question 5:** There is not any table containing the dataset and your description about the columns and rows you used in your research.

**Response:** The table containing the dataset description has been added (refer table 1).

**Question 6:** The discussion part is too short.

**Response:** More information about model performance based on MSE and RMSE is added in discussion

**Question 7:** May we have any future work regarding omicron? what would you do in the future about it?

**Response:** Future work is mentioned in the conclusion and highlighted for your reference
